# A 3D-printed modular magnetic digital microfluidic architecture for on-demand bioanalysis

**DOI:** 10.1038/s41378-020-0152-4

**Published:** 2020-06-29

**Authors:** Pojchanun Kanitthamniyom, Aiwu Zhou, Shilun Feng, Aiqun Liu, Shawn Vasoo, Yi Zhang

**Affiliations:** 1 Singapore Centre for 3D Printing, School of Mechanical and Aerospace Engineering, Nanyang Technological University, Singapore, Singapore; 2 School of Mechanical and Aerospace Engineering, Nanyang Technological University, Singapore, Singapore; 3 School of Electrical and Electronic Engineering, Nanyang Technological University, Singapore, Singapore; 4 National Center for Infectious Disease, Tan Tock Seng Hospital, Singapore, Singapore

**Keywords:** Microfluidics, Biosensors

## Abstract

Magnetic digital microfluidics (MDM) manipulates fluids in the form of droplets on an open substrate, and incorporates surface energy traps (SETs) to facilitate the droplet manipulation. Conventional MDM devices are fabricated monolithically, which makes it difficult to modify the device configuration without completely overhauling the original design. In this paper, we present a modular MDM architecture that enables rapid on-demand configuration and re-configuration of MDM platforms for customized bioanalyses. Each modular component contains a SET and a Lego-like antistud that fits onto a base board with Lego-like studs. We illustrate the versatility of the modular MDM architecture in biomarker sensing, pathogen identification, antibiotic resistance determination, and biochemical quantification by demonstrating immunoassays, phenotypical assays and enzymatic assays on various modular MDM platforms configured on demand to accomplish the fluidic operations required by assorted bioanalytical assays. The modular MDM architecture promises great potential for point-of-care diagnostics by offering on-demand customization of testing platforms for various categories of diagnostic assays. It also provides a new avenue for microfluidic assay development with its high configurability which would significantly reduce the time and cost of the development cycle.

## Microsystems & Nanoengineering: Magnetic digital microfluidics: Customized bioanalyses

A Lego-inspired, modular magnetic digital microfluidic architecture enables customizable bioanalysis. Magnetic digital microfluidics controls droplets on a surface via magnetic particles, with the liquid droplets themselves acting as mini bioreactors for analysis. Such devices often include surface energy traps to aide droplet manipulation. However, because they are fabricated as monolithic systems, they cannot be altered for different bioanalysis post-fabrication. Here, a team led by Yi Zhang from Nanyang Technological University, Singapore, reports a modular design for magnetic digital microfluidic devices, in which functional modules are configured and reconfigured via Lego-like studs. This modular design allows users to build testing platform on demand for a wide range of bioanalyses.

## Introduction

Magnetic digital microfluidic (MDM) employs magnetic particles to manipulate a small volume of liquid on an open surface, where the liquid forms droplets that function as self-contained bioreaction chambers^1^. MDM technology has been widely adopted for molecular diagnostics^2–8^, immunosensing^9^, antimicrobial susceptibility testing^10,11^, and other bioanalyses^12–14^. Besides the advantages common to all microfluidic systems, such as low reagent consumption and integrated analysis^15,16^, MDM system offers additional benefits with its simple and flexible fluidic operation^1^. Many MDM platforms incorporate surface energy traps (SETs)—surface topographical structures or chemical modifications—to facilitate the droplet manipulation^3,5^. SETs are critical components required to accomplish intricate fluidic operations on MDM platforms.

Owing to monolithic fabrication techniques, the design philosophy of MDM systems places a strong emphasis on the high-level integration in order to achieve micro total analysis (microTAS) on a lab-on-a-chip (LOC) platform^17–19^. As a result, each MDM design is tailored to a highly specialized application. Consequently, any change in device configuration, no matter how small, would entail a complete overhaul of the original design, which incurs substantial costs and time. One solution to this problem is through modularization. Instead of fabricating all microfluidic components on one single chip, the microfluidic network is constructed by assembling discrete modules sculped with basic microfluidic elements via standardized interconnects^20–28^. The modularization expedites design cycles and empowers customized microfluidics with its plug-and-play capability. Nevertheless, modularized continuous-flow microfluidic systems are frequently beset by the leakage problem at the interconnect. In contrast, MDM could take the full advantage of modularization without being concerned by the leakage because of the discrete nature of digital microfluidics.

In this work, we propose a modular MDM architecture that can be customized on demand into any arbitrary configurations to meet the requirements of assorted bioanalytical assays. Each modular block has a SET feature on one side and a Lego-like antistud on the other side. These modular blocks are easily press-fit onto the base board with Lego-like studs to form an MDM fluidic network. All modular components are fabricated with additive manufacturing techniques, which allows the incorporation of complex geometries into the design. This modular architecture significantly lowers the barriers to adopting MDM technology, allowing end users to program customized modular MDM platforms to realize fluidic operations required by bioanalytical assays. This modularization approach is particularly appealing for assay development and on-demand point-of-care testing. For instance, if the user finds that an additional procedure is coveted in a new assay under development, one additional MDM module can be easily introduced into the MDM fluidic network without much added cost. In the scenario of point-of-care diagnostics, once the healthcare provider decides which tests are required, MDM testing platforms can be assembly on demand accordingly.

## Results

### Modular SET design concept

**Fig. 1:**
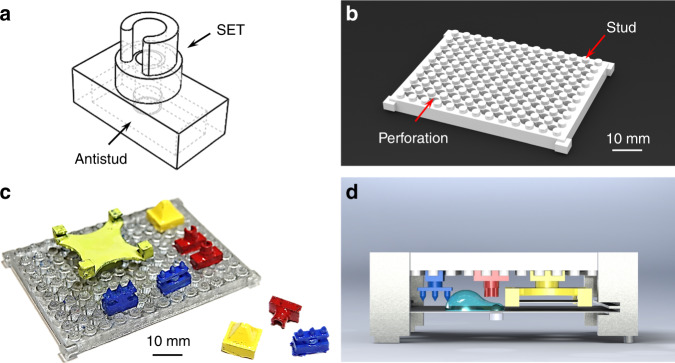
Modular MDM architecture. **a** A representative modular component for particle extraction. **b** Illustration of the base board. **c** A modular MDM platform constructed with modular components on the base board. The modular components are painted to aid the visualization. **d** Illustration of a complete modular MDM system

Individual modular components are assembled onto the base board via standardized Lego-style studs and antistuds (Fig. 1). Each modular component contains an antistud and a functional SET element for MDM operations, such as particle extraction, liquid dispensing, and passive mixing. A representative modular component for particle extraction is shown in Fig. 1a. This modular component is rendered hydrophobic with a Teflon AF coating, but the circular tip is coated with polydopamine, resulting in a high surface energy to anchor the droplet^9,29^. A standard library of MDM modular components is shown in Supplementary Fig. S1. The base board (Fig. S1a) contains an array of studs, and the space between studs is perforated to create access for the liquid dispenser (Fig. 1b). The locations of the perforated holes are aligned. Hence, the locations of the droplets dispensed through these perforated holes are well defined. MDM fluidic networks are constructed by press-fitting SET modules onto the base board (Fig. 1c. The modular components are color painted to aid visualization. Actual experiments are done with unpainted parts.). These modular components could be arranged in any arbitrary configurations depending on the fluidic operations required by the bioanalytical assays. Thanks to the modular architecture, MDM platforms can be quickly reconfigured by removing existing modular components, introducing new modular components, or swapping modular components of different functions, in a way analogous to movable typesetting in printing. From developers’ perspective, the design and fabrication of modular MDM devices are relatively simple because there is no need to be concerned by the system-level integration. From end users’ perspective, the modularization enables customized design, rapid prototyping, and on-demand construction of MDM platforms for a wide range of bioanalytical assays commonly performed in clinical settings.

To manipulate droplets, the assembled modular MDM device is positioned above a piece of Teflon-coated coverslip (Fig. 1d). Both the base board and the glass coverslip (Fig. S1b) are locked to a support frame (Fig. S1c). Droplets are sandwiched between the modular components and the glass coverslip, forming a two-plate MDM configuration. In regions without the modular component, the droplet is only in contact with the glass coverslip and moves smoothly. Once being moved to the region with modular components, the droplet interacts with the SETs for a diverse range of fluidic operations. The height of the modular block is adjusted according to the droplet height to ensure contact between the droplet and the SETs (see Supplementary Fig. S2 for the relationship between the droplet height and volume). A permanent magnet attached to a motorized translational stage is stationed beneath the glass coverslip to control the motion of droplets via magnetic particles added to the droplet.

### Fluidic operation with modular MDM

Basic droplet-based fluidic manipulations are demonstrated on the modular MDM platform.

**Fig. 2:**
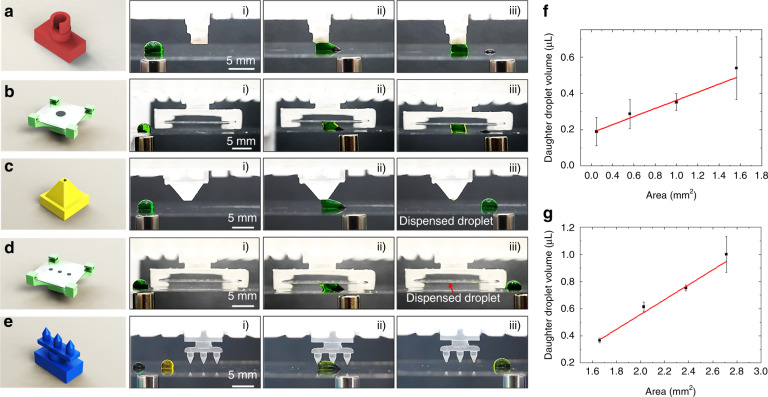
Demonstration of droplet manipulation with modular SET components. **a** Particle extraction module. **b** Particle extraction using SETs on glass incorporated via the holder module. **c** Liquid dispensing module. **d** Liquid dispensing using SETs on glass incorporated through the holder module. **e** Passive mixing module. **f** Volume of daughter droplets vs the area of SET on the liquid dispensing module. **g** Volume of daughter droplets vs the area of SET on glass incorporated via the holder module

Particle extraction is an important fluidic operation to separate the solid phase from the liquid phase in heterogeneous assays. The particle extraction module comprises of a hollow cylinder with its circular tip coated with polydopamine (Fig. S1d). As the magnet drags the droplet through the particle extraction module, the entire droplet is anchored to the SET while the magnetic particle cluster continues moving forward until it breaks from the droplet (Fig. 2a and Video S1). In addition to creating SETs directly on 3D-printed modular blocks, SETs can also be created on the glass coverslip and incorporated into the modular MDM platform via a holder module (Fig. S1e) in a configuration shown in Fig. 2b. The SETs are fabricated by dispensing dopamine monomer solution on the Teflon-coated glass coverslip, and the polydopamine synthesized in situ forms a region with a high surface energy on the glass coverslip to immobilize the droplets. In this case, droplets are sandwiched between two pieces of glass coverslip, and the particle extraction is accomplished with SETs on the top glass coverslip (Fig. 2b and Video S2).

Liquid dispensing is another important fluidic operation for making aliquots and serial dilution on the MDM platform by taking a small volume from a large volume of droplet^5,10^. The liquid dispensing module comprises of a pyramid with a small flat tip coated with polydopamine (Fig. S1f). Due to the small area of the SET, only a small portion of the droplet is attached to the SET. As the magnet continues pulling the droplet forward, the droplet necking point around the SET would break, dispensing a small daughter droplet (Fig. 2c and Video S3). The SETs for liquid dispensing could also be incorporated into the modular MDM platform via the holder module (Fig. 2d and Video S4). The holder module enables us to transfer droplets from one platform to another by sliding the glass coverslip in and out of the holder module from the side without having to disassemble the platform. The dispensed droplets could be subsequently transferred to another MDM platform with the glass coverslip. The volume of the daughter droplets generated using SETs on the 3D-printed modular component (Fig. 2c) and SETs on the glass coverslip (Fig. 2d) are plotted vs the area of SETs in Fig. 2f, g, respectively. In both scenarios, the volume of the daughter droplets is proportional to the area of the SETs, which is in agreement with our previous study^9^. It is noted that the daughter droplets generated directly on the 3D-printed modular block have a larger variability with a CV of 6–8% for small daughter droplets (up to 0.75 μL) and 17% for large daughter droplets (Fig. 2f). The daughter droplets generated by SETs on the glass coverslip show a smaller variability with a CV of 3–6% for small daughter droplets and 13% for large daughter droplets (Fig. 2g). The larger variability on 3D-printed modular block is likely due to the polydopamine coating. Unlike a flat surface on which the size of the polydopamine-coated SETs can be precisely controlled, the polydopamine coating on the irregular surface of the 3D-printed modules is difficult to control. Compared to our previous study^5,9^, the overall large variability possibly results from the two-plate MDM configuration. Unlike the single-plate configuration where the magnetic force and the surface tension of SETs are coaxial, the two forces are on two different planes in the two-plate configuration, which complicates the droplet splitting mechanism. As the SETs are getting larger, the droplet operation changes from liquid dispensing to particle extraction. At the boundary of the two operations, the volume of the dispensed droplet tends to vary. Therefore, to dispense relatively uniform droplets, it is better to use the holder module with small SETs for liquid dispensing or use single-plate configuration by fabricating the SETs on the bottom substrate.

**Fig. 3:**
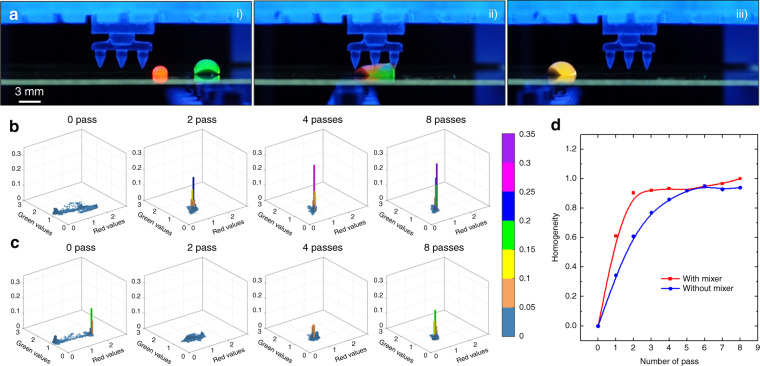
Droplet mixing with the mixing module. **a** Droplet behavior as it goes through the mixing module. **b**, **c** 2D histogram of droplet pixels in red–green color space. A single sharp peak indicates a high mixing homogeneity. **b** with mixer, **c** without mixer. **d** Mixing homogeneity vs number of pass

We have created a passive mixing module to facilitate mixing inside droplets (Fig. 2e, and Video S5). Compared to previously reported active droplet mixer^30^, the passive mixer is easier to implement. The mixing module consists of three tapered pillars with a rough surface resulted from the 3D printing process (Fig. S1g). Although the surface of the pillars is coated with Teflon, the droplet would still adhere slightly to the pillar due to the Wenzel wetting on the rough surface. The droplet is stretched by the magnetic force and the adhesive force as it moves through the mixing module until a sudden release of the droplet from the pillar causes the droplet to rebound, which induces mixing inside the droplet via a “sling” effect. Because of the small contact area between the droplet and the tapered pillar, only a negligible amount of liquid is withheld on the pillar. We have assessed the mixing effectiveness inside droplets on the modular MDM platform with and without the mixing module (Fig. 3a and Video S6). A droplet containing quantum dot Qdot 525 is dragged by magnetic particles to merge with another droplet containing quantum dot Qdot 605. The two quantum dots are mixed by moving the droplet back and forth on the glass substrate. The fluorescent images of the droplet after each pass are analyzed to assess the mixing effectiveness. The normalized pixel intensities from the red and green channels of each image are plotted in a 2D histogram where the *x*-axis represents normalized red intensity, the *y*-axis represents normalized green intensity, and the *z*-axis indicates the proportion of pixels with a particular red–green combination. Right after the two quantum dots merge, they largely remain separate, which manifests as two isolated clusters in the 2D histogram (Fig. 3b, c). As the two quantum dot solutions mix, the two clusters in the 2D histogram would collapse into a single one, and the peak width of the cluster signifies the mixing homogeneity. The sharper the peak gets, the more homogeneous the solution inside the droplet becomes. In the event with the mixing module (Fig. 3b), the two clusters merge into one with an evident sharp peak after only 2 passes, indicating rapid homogeneous mixing. In contrast, in the event without the mixing module (Fig. 3c), although the two clusters merge into one after 2 passes, the pixels are widely spread in the red–green color space without a distinct peak, suggesting a poor mixing homogeneity. A single peak appears only after the 4th pass and exhibits a broad distribution in the red–green color space, suggestion ineffective mixing. The mixing homogeneity is plotted vs the number of pass (Fig. 3d), which shows that the droplet becomes homogeneous more rapidly with the mixing module. Only 2 passes are required to achieve a high homogeneity with the mixing module, whereas it requires at least 5 passes to reach the same level of homogeneity when the mixer is not in use.

### Bioanalysis with on-demand modular MDM platform

In real-world clinical settings, oftentimes several categories of bioanalytical assays are required to diagnose and prognose a clinical condition. For instance, in the case of infectious disease management, enzyme-linked immunosorbent assay (ELISA) is often used to detect antigens and inflammation markers in order to confirm the infection and test for sepsis. Subsequently, phenotypical assays are usually required to identify the pathogen and measure the antimicrobial susceptibility. Blood biochemical parameters are also assessed with enzymatic assays to monitor patient’s health status. These bioanalytical assays entail different fluidic operations depending on the variety of reagents used and the complexity of the assay procedure. There is no single device that fits all purposes. Fortunately, with our modular architecture, one can construct MDM platforms on-demand using standard MDM modular components to perform assorted assays required for disease diagnosis and prognosis. In this section, we demonstrate three categories of bioanalytical assays on our modular MDM platforms.

### ELISA for detection of biomarkers

**Fig. 4:**
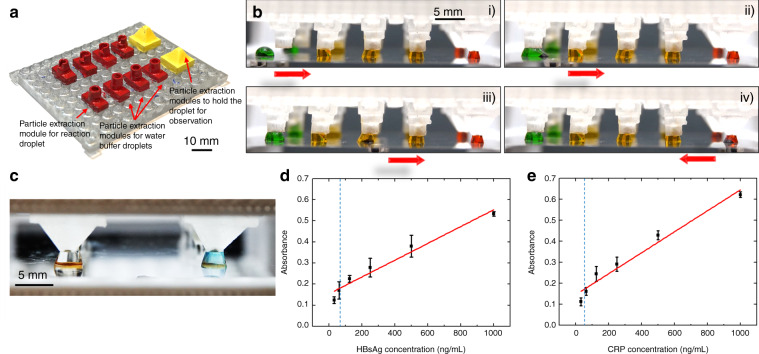
ELISA on modular MDM platform. **a** Modular MDM device configured for ELISA. **b** Droplet operation procedure for ELISA on the modular MDM device. **c** A representative picture of the reaction droplets. Positive reaction droplet appears blue and the control droplet appears clear. **d** Standard curve of HBsAg measured on the modular MDM platform. **e** Standard curve of CRP measured on the modular MDM platform. The dash lines indicate the limit of detection

Two ELISAs are conducted to detect hepatitis B surface antigen (HBsAg) and C-reactive protein (CRP). HBsAg is a diagnostic marker for hepatitis B viral infection. CRP is an inflammatory biomarker, and a high level of CRP in the blood may be a sign of sepsis caused by severe infections. Two types of particle extraction modules (shown in red and yellow in Fig. 4a) are used for different purposes. Four red modules are used in the washing zones, and one yellow module is used in the observation zone. Although the shape of the yellow module resembles the liquid dispensing module, the size of the SET is increased for particle extraction. Two replicates are conducted in parallel with two sets of modular components arranged in the configuration shown in Fig. 4a. The magnetic particles function both as the solid substrate for molecule immobilization and as the magnetic actuator for droplet manipulation. We demonstrate the ELISA process with false-color droplets (Fig. 4b and Video S7). The magnetic particles conjugated with the capture antibody is incubated with the target protein and the horseradish peroxidase (HRP)-labeled detection antibody in the sample droplet (green). After incubation, the magnetic particles are extracted from the sample droplet and moved through three washing buffer droplets (orange) for washing. A number of particle extractions are performed with the assistance of the particle extraction modules during this process. After washing, the magnetic particles with sandwiched antibody-antigen on the surface are merged with the droplet containing enzyme substrate for development (red). In the end, the magnetic particles are removed from the last droplet for easy visualization. When running ELISA, the last droplet turns blue if the sample contains the target protein, and remains clear if it does not (Fig. 4c). The absorbance of the developed substrate in the last droplet is plotted as a function of the target protein concentration (Figs. 4d, e). Both HBsAg and CRP display a good linear correlation between the target protein concentration and the absorbance within the testing range (31–1000 ng/mL). The limit of detection for HBsAg is 61.6 ng/mL on the modular MDM platform, close to the limit of detection of 55.5 ng/mL of the benchmark performed in the microwell plate (Fig. S3a). The limit of detection for CRP is 59.8 ng/mL on the modular MDM platform, similar to the limit of detection of 28.8 ng/mL of the benchmark performed in the microwell plate (Fig. S3b).

### Phenotypic assay for pathogen sensing and antibiotic resistance determination

**Fig. 5:**
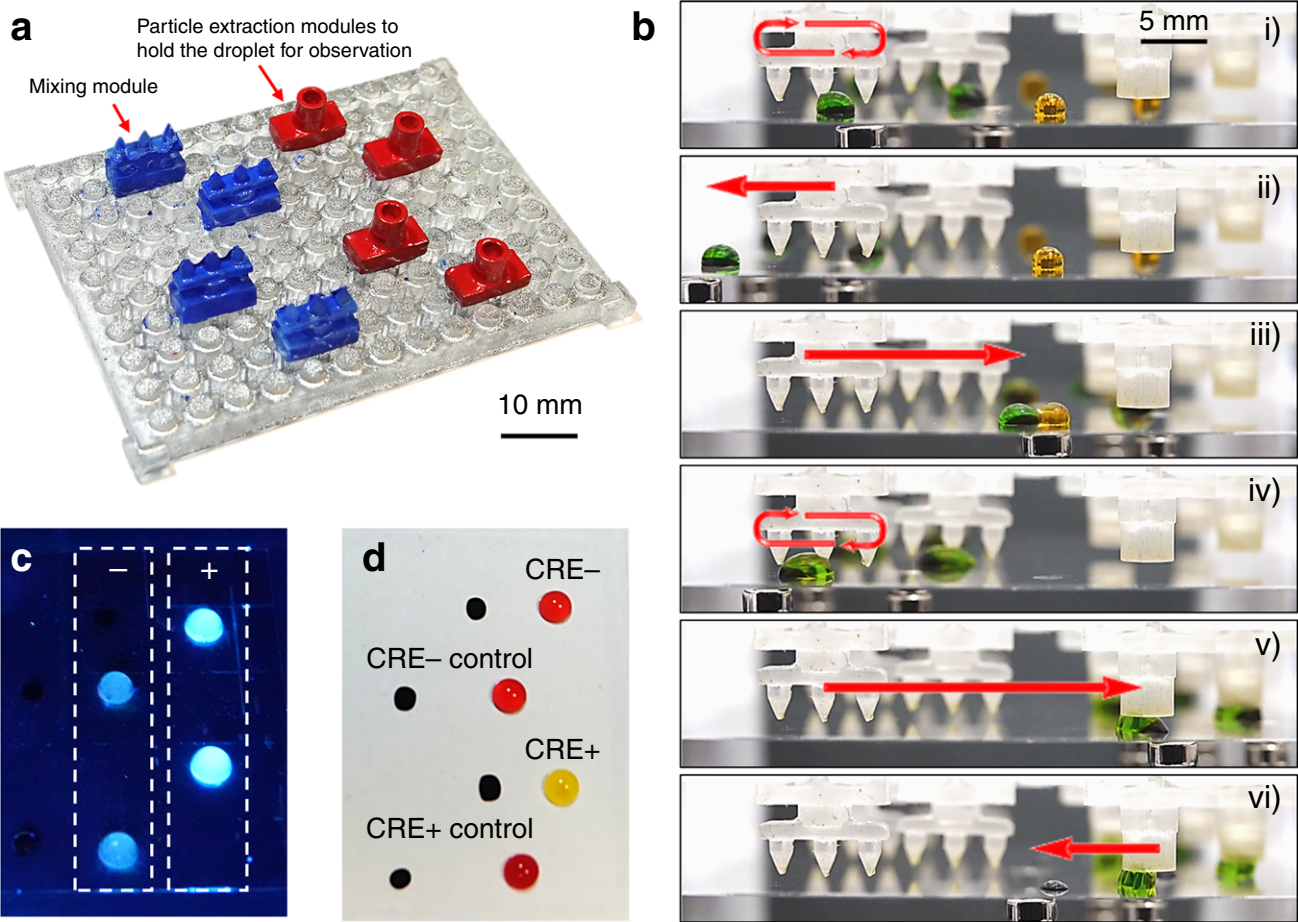
Phenotypical assay on modular MDM platform. **a** Modular MDM device configured for phenotypical assays. **b** Droplet operation procedure for phenotypical assays on the modular MDM device. **c** Result of MUG test on the modular MDM platform for the detection of *E. coli*. **d** Result of Carba NP test on the modular MDM platform for the determination of drug resistance against carbapenem. Reaction droplet with CRE+ strain turns yellow, and the reaction droplet with CRE- strain remains red. Two control reaction droplets stay red, indicating a valid test

An MDM platform is configured (Fig. 5a) to detect the presence of *E. coli* and determine its resistance against carbapenem, a group of last-line antibiotics that fights Gram-negative bacterial infections using two phenotypical assays^31^. The presence of *E. coli* is detected by measuring the activity of beta-glucuronidase produced by the bacteria (MUG test)^32^. The antibiotic resistance against carbapenem is determined by measuring the pH change as a result of the hydrolysis of carbapenem induced by carbapenemase, the enzyme produced by carbapenem-resistant *Enterobacteriaceae* (CRE) (Carba NP test)^33,34^. Each phenotypic assay requires one mixing module and one particle extraction module. Four sets of modular components are arranged for samples and controls (Fig. 5a). For both assays, the bacteria are first added to the lysis buffer droplet (green) and mixed by moving back and forth through the mixing module to promote bacteria lysis (Fig. 5b and Video S8). The sample droplet is then merged with the reaction buffer droplet (orange) and mixed with the mixing module. Lastly, the reaction droplet is anchored to the particle extraction module in the observation zone. The magnetic particles are removed from the droplet for easy visualization.

In the case of *E. coli* detection, the reaction buffer droplet contains a fluorogenic enzyme substrate 4-methylumbelliferyl-beta-D-glucuronide (MUG). The beta-glucuronidase produced by *E. coli* cleaves MUG, generating a blue fluorescent signal. The reaction droplets are observed under the 365-nm UV illumination. Tests are run in duplicates with a sample and a control in each test. Samples containing *E. coli* display a strong blue fluorescent signal (Fig. 5c). In contrast, the control reaction without *E. coli* only display a weak background. The benchmark reactions performed in microwell plate show the same results (Fig. S4a).

To determine the antibiotic resistance, the lysed bacteria are merged with the reaction buffer droplet that contains imipenem and a pH indicator. If the bacterial strain is CRE+ (resistant), the imipenem is hydrolyzed, which reduces the buffer pH and changes the color of the droplet to yellow (Fig. 5d). In contrast, if the bacterial strain is CRE− (susceptible), the color of the droplet remains red. A control reaction is performed with each bacterial sample. In the control reaction, bacteria are added to the sample droplets, but no imipenem is included in the reaction buffer droplet. For the Carba NP assay to be valid, the color of the control droplets must remain red throughout the reaction. The same results are observed in the benchmark reactions performed in microwell plate (Fig. S4b).

### Enzymatic assay for protein and glucose sensing

The sensing of biochemical parameters, including protein concentration and glucose concentration, using enzymatic assays is demonstrated on the modular MDM platforms.

**Fig. 6:**
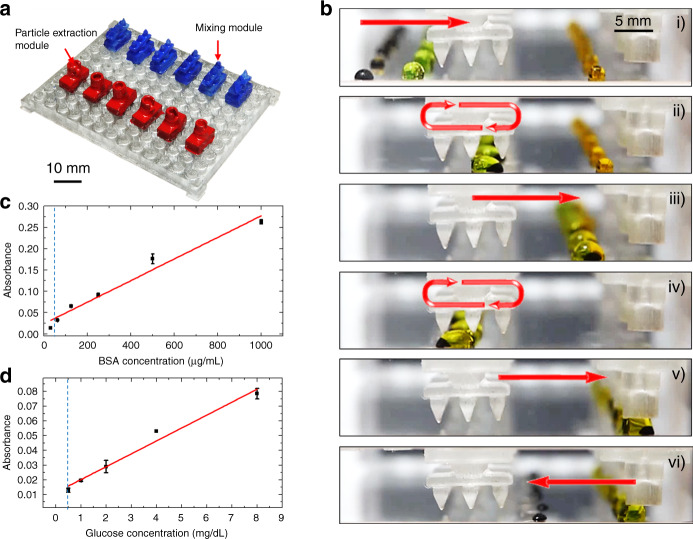
Enzymatic assay on modular MDM platform. **a** Modular MDM device configured for enzymatic assays. **b** Droplet operation procedure for enzymatic assays on the modular MDM device. **c** Standard curve of BSA measured on the modular MDM platform. **d** Standard curve of glucose measured on the modular MDM platform. The dash lines indicate the limit of detection

Protein concentration is measured using the bicinchoninic acid assay (BCA). Strictly speaking it is not an enzymatic assay, but in is included in this section due to its similar assay procedure. Each droplet-based BCA requires one mixing module and one particle extraction module. Six sets of modular components are arranged in parallel to perform six reactions concurrently (Fig. 6a). Magnetic particles are added to the sample droplets (black) containing bovine serum albumin (BSA). The concentration of BSA in these 6 droplets form a serial dilution. Next, the sample droplets are moved in parallel to merge with the working solution droplets (green) (Fig. 6b and Video S9). The merged droplets are mixed by going through the mixing modules back and forth, and subsequently incubated for 15 min. After incubation, the assay droplets are merged with the stop-solution droplets (orange) and mixed a few times under the mixing modules before being moved to the observation zone. The particle extraction modules at the observation zone hold the droplet in position while the magnetic particles split from the droplet. The absorbance of the reaction droplets has a good linear relationship with the BSA concentration within the testing range (31–1000 μg/mL) (Fig. 6c). The limit of detection is 54.6 μg/mL on the modular MDM platform. The benchmark reaction performed in the microwell plate has a limit of detection of 27.4 μg/mL (Fig. S5a).

Glucose concentration is measured on the modular MDM platform with the same parallel droplet configuration and operation procedure. The absorbance of the reaction droplets shows a good linear relationship with glucose concentration within the testing range (0.5–8 mg/dL) (Fig. 6d). The limits of detection are comparable between the modular MDM platform and the microwell plate benchmark, which are 0.47 and 0.35 mg/dL, respectively.

## Discussion

In this work, we have designed a modular architecture that empowers on-demand construction of MDM platforms for a variety of bioanalyses, ranging from biomarker detection, pathogen identification, antibiotic resistance determination, and biochemical quantification. The versatility of modular MDM platforms has been aptly illustrated with several categories of bioanalytical assays, including immunoassays, phenotypical assays, and enzymatic assays. Both quantitative and qualitative assessments are on the modular MDM platforms, and their performances are comparable to the benchmark reactions performed in the microwell plate.

To design a modular MDM platform for a particular bioanalytical assay, the first step is to identify the type and number of fluidic operations. Each fluid addition requires a droplet merging operation by moving one droplet to merge with another. Each separation of liquid phase from solid phase requires a particle extraction module. Each mixing step requires a mixing module. All required MDM modules are then arranged on the base board, and the droplets are programed to move through these MDM modules in a particular order according to the assay procedure. By doing so, we are able to translate many bioanalytical assays to the modular MDM platform and construct these platforms on demand.

The modular architecture uses Lego-like stud-antistud fitting for rapid MDM device configuration and reconfiguration. The library of standard SET modular components provides a versatile toolbox at our disposal. As a result of these advantages, modular MDM systems promise great potential in point-of-care diagnostic scenarios in which the required tests cannot be decided a priori, or several types of tests are needed on site. In these scenarios, modular MDM platforms can be constructed on demand to accomplish the fluidic operations entailed by these diagnostic tests with a low cost and a rapid turnaround time. Although the measurements were done off chip in this study, colorimetric readouts could be taken by direct visualization with unaided eyes or with cell phone-based imaging analysis systems for point-of-care testing^35^. Modular MDM systems also provide a promising avenue for rapid assay development. Although we only demonstrate analytical assays in the current study, we believe that modular MDM system is also applicable to preparative assays, chemical synthesis, and material production by incorporating other functional modules such as droplet compatible temperature controller for incubation and thermal cycling^2,36^. Any change in assay procedures could be rapidly implemented by reconfiguring the affected zones while keeping the rest zones of the modular MDM platform untouched. Doing so enables rapid device prototyping and assay assessment, which would significantly reduce the time and cost of the development cycle.

## Methods and materials

### Device design and fabrication

All designs were performed in SolidWorks (Dassault Systèmes) and fabricated with 3D printing. The base board and all the modular components were printed with clear resins using stereolithography (SLA), and the support holder was printed with acrylonitrile butadiene styrene (ABS) using fused deposition modeling (FMD). The 67 mm × 50 mm base board contained 13 × 10 studs with a diameter of 3 mm each. The spaces between studs were perforated to create access for the liquid dispenser. Droplets were dispensed through the perforated holes, and the location of these perforated holes provides reference for droplet alignment. The particle extraction module also contained a hole in the middle, and droplets could be dispensed directly through the hole and immobilized under the module. The magnets for droplet manipulation were programmed to move according to the location of the holes through which the droplets are dispensed. The modular components contained antistuds of two different sizes. The 10 mm × 10 mm antistud fit into four studs (2 × 2), and the 10 mm × 5 mm antistud fit into two studs (1 × 2). The modular components were assembled onto the base board by press fitting.

All modular components were dip coated with 1% Teflon AF solution in FC-40 (DuPont) at room temperature. The tip of the particle extraction module and the liquid dispensing module was dip coated with polydopamine with dopamine-HCL solution of 5 mg/mL in 10 mM Tris buffer, pH 8, for 3 h followed by rinsing with distilled water.

The glass coverslips (Grade No. 1, Thermofisher Scientific) were spin-coated with 1% Teflon AF solution at 500 rpm for 40 s, followed by baking at 120 °C for 2 min. SETs were patterned on the Teflon-coated coverslip with polydopamine using the protocol presenter earlier^9,37^. To briefly recap, dopamine-HCl monomer is dissolved in Tris buffer at pH = 8 with a final concentration of 2 mg/mL. A dopamine monomer solution is dispensed on the Teflon-coated glass coverslip and incubated for 1 h in a humid chamber. After incubation, the glass coverslip is rinsed with water and dried before use.

### Magnetic control and droplet manipulation

The droplets were manipulated using either silica-passivated magnetic particles (Qiagen Magattract suspension G) or streptavidin-coated magnetic particles (Life Technologies Dynabeads M-270). All experiments except ELISA were run with the silica-passivated magnetic particles. NdFeB permanents magnets of 5 mm × 5 mm (Diameter × Height) were used to control the droplet motion. In all, 3 mm × 2 mm (Diameter × Height) permanent magnets were used in the magnet array for 6-droplet parallel control. The strength of the two types of magnets were 159 and 78 mT, respectively. The minimum volume tested was 2 μL (the volume of magnetic particles needs to be adjusted accordingly), and the maximum volume is about 25 μL, which is limited by the height between the glass substrate and the modules.

False-color droplets were colored with food dye to aid visualization. For experiments shown in Fig. 2, each sample contained 10 μL of water and 7 μL of magnetic particles. The motion of the permanent magnets was controlled by an automated 2-axis linear translational stage moving at a speed of 300 mm/min.

The volume of the dispensed daughter droplets was estimated using the approach reported earlier. In all, 10 μL of water was added to the daughter droplets split from a parent droplet containing fluorescein. The volume of the daughter droplet was calculated according to^30^

1 $$V_d = \frac{{C_f}}{{C_i - C_f}}V_{{\mathrm{H}_2{\mathrm{O}}}}$$

where ***C***_***f***_ was the final fluorescein concentration of the daughter droplet after water addition, and ***C***_***i***_ was the initial fluorescein concentration of the parent droplet, ***V***_***d***_ is the volume of the daughter droplet and $V_{{\mathrm{H}_2{\rm O}}}$ is the volume of the added water, which is 10 μL in this case.

### Analysis of mixing in droplets

Mixing in droplets were performed both with and without the mixing module. A droplet containing 20 μL of fluorescein and 7 μL of magnetic particles were merged with a 5-μL droplet containing quantum dot Qdot 605. The hydrodynamic radii of Qdot 605 and Qdot 525 are 14 ± 3 nm and 8 ± 3 nm, respectively. The average diffusion coefficient of Qdot 605 and Qdot 525 are 1.7 × 10^−11^ and 3.6 × 10^−11^ m^2^/s, respectively^38^. They were treated as fluorescent dyes instead of particles^39,40^. The droplets were illuminated with a 365-nm UV lamp, and the fluorescent images of the droplet were taken after each pass.

The mixing homogeneity was calculated according to^30^

2 $$H = 1 - \frac{{M_c - M_f}}{{M_i - M_f}}$$

where ***H*** was the mixing homogeneity, and ***M***_***c***_, ***M***_***i***_, and ***M***_***f***_ were the mixing indices of the current, initial and final pass, respectively.

The mixing index was calculated by calculating the variance of pixel intensity in the red and green channels of each fluorescent image according to

3 $$M = \sqrt {\frac{1}{N}\mathop {\sum }\limits_{i = 1}^N \left( {\left( {I_{Gi} - \bar I_G} \right)^2 + \left( {I_{Ri} - \bar I_R} \right)^2} \right)}$$

where ***M*** was the mixing index, ***N*** was the total number of pixels, ***I***_***G***_ and ***I***_***R***_ were the pixel intensity in green and red channels of each pixel, and ${\bar{\boldsymbol I}}_{\boldsymbol{G}}$and ${\bar{\boldsymbol I}}_{\boldsymbol{R}}$ were the mean pixel intensity in the green and red channels. The background and bright spot due to reflection were removed by setting a grayscale threshold. The normalized intensities were plotted in a normalized 2D histogram.

### Bioanalytical assays on MDM platforms

#### ELISA

Droplet-based ELISA was used on the modular MDM platform. The capture antibodies (HBsAg antibodies from Arista Biologicals, CRP antibodies from Abcam) were labeled with biotin using biotinylation kit (Abcam) following the manufacturer’s instruction. The detector antibodies were labeled with horseradish peroxidase (HRP) using HRP labeling kit (Abcam) following the manufacturer’s instruction. The biotinylated capture antibodies were conjugated to streptavidin-labeled magnetic beads (Dynabeads M270 from Life Technologies). The detector antibody was mixed with the target (recombinant HBsAg from Arista Biologicals and recombinant CRP from Abcam), and the final detector antibody concentration was 0.5 μg/mL. The sample droplet containing 10 μL capture antibody-labeled magnetic beads and 10 μL of target-detector antibody mixture was incubated for 1 hour. After incubation, the magnetic beads were moved through three 15-μL droplets of washing buffer held in place by the particle extraction modules. After washing, the magnetic beads with sandwiched antibody-antigen structure on the surface were incubated inside a 10-μL 3,3′,5,5′-tetramethylbenzidine (TMB) droplet for 30 min. Last, the magnetic beads were removed from the TMB droplet with the assistance of the particle extraction module.

#### *E. coli* sensing

*E. coli* produces beta-glucuronidase, which hydrolyzes the substrate 4-methylumbelliferyl-beta-d-glucuronide (MUG), resulting in blue fluorescence under the UV illumination. In all, 10 μL 10^6^ CFU *E. coli* in suspension culture was mixed with a 10-μL droplet containing 0.1 mg/ml lysosome. In all, 4 μL of magnetic particles were added to the sample droplet. After going through the mixing module back and forth, the sample droplet was merged with a 10-μL droplet containing 0.35 mg/mL MUG (Sigma Aldrich) and mixed by going through the mixing module. After the magnetic particles were removed with the assistance of the particle extraction module, the droplet was incubated for 30 min at the observation zone, and the fluorescent signals were observed under the illumination of a 365-nm UV light source.

#### Antibiotic resistance measurement

Antibiotic resistance against carbapenem was determined using the Carba NP assay. The CRE+ (resistant) strain hydrolyzed the antibiotics and reduced the buffer pH, changing the color of the solution from red to yellow. For each bacterial strain, two reactions were carried out. One of the reactions contained 6 mg/mL imipenem (USP) in the reaction buffer, and the other one did not. The reaction buffer comprised of 0.5% (w/v) red phenol and 10 mM zinc sulfate (buffered to pH 7.8 by adding 0.1 N NaOH). To perform Carba NP, a bacterial colony was first added to a 10-μL lysis buffer (10% B-PERII, Thermo Scientific) that contained 4 μL of magnetic particles. The sample droplet was then mixed by moving through the mixing module back and forth to promote the lysis. After mixing, the sample droplet was merged with a 10-μL reaction buffer droplet, and the merged reaction droplet was mixed again. The final reaction droplet was incubated for 30 min before observation.

#### Protein quantification

Protein quantification was conducted using Pierce™ Rapid Gold BCA Protein Assays (Thermofisher Scientific) by downscaling the assay volume. The sample droplet contained 3 μL BSA solution droplet and 4 μL magnetic particles. The sample droplet was moved to merge with a 15-μL droplet of working solution. The merged reaction droplet was mixed by going through the mixing module back and forth before being incubated for 30 min. After incubation, the reaction droplet was merged with a stop buffer droplet containing 7.5 μL of 1 M HCl and mixed by going through the mixing module twice. The absorbance was measured with a micro UV-Vis spectrometer. The absorbance at 480 nm was plotted as a function of the target concentration.

#### Glucose quantification

Glucose quantification was conducted using glucose colorimetric detection kit (Invitrogen) following the manufacturer’s instruction. The sample droplet was moved to merge with a 5-μL droplet of glucose oxidase solution. The merged reaction droplet was mixed by going through the mixing module back and forth. After mixing, the reaction droplet was merged with a 5-μL droplet containing 1x HRP and the enzyme substrate. The reaction droplet was incubated for 30 min before observation. The absorbance was measured with a micro UV-Vis spectrometer. The absorbance at 560 nm was plotted as a function of target concentration.

## Supplementary information

Video S1

Video S2

Video S3

Video S4

Video S5

Video S6

Video S7

Video S8

Video S9

Supplementary Information
