# Supplementary material for: A 3D-printed modular magnetic digital microfluidic architecture for on-demand bioanalysis
Source: Microsyst Nanoeng. 2020 Jun 29;6:48. doi: 10.1038/s41378-020-0152-4 (PMC8433373; doi:10.1038/s41378-020-0152-4)
Supplement: Supplementary file 10 — Supplementary Information [file 41378_2020_152_MOESM10_ESM.docx]

**A 3D-Printed Modular Magnetic Digital Microfluidic Architecture for On-Demand Bioanalysis**

Pojchanun Kanitthamniyom^1^, Aiwu Zhou^2^, Shilun Feng^3^, Aiqun Liu^3^, Shawn Vasoo^4^, Yi Zhang^1^*

^1^Singapore Centre for 3D Printing, School of Mechanical and Aerospace Engineering, Nanyang Technological University

^2^School of Mechanical and Aerospace Engineering, Nanyang Technological University

^3^School of Electrical and Electronic Engineering, Nanyang Technological University

^4^National Center for Infectious Disease, Tan Tock Seng Hospital

Correspondence to Yi Zhang ([yi_zhang@ntu.edu.sg](mailto:yi_zhang@ntu.edu.sg)), Tel: +65-67905590, 50 Nanyang Avenue, Singapore 639798, Singapore

All videos can be downloaded from any of the following link.

Onedrive:

<https://entuedu-my.sharepoint.com/:f:/g/personal/yi_zhang_staff_main_ntu_edu_sg/EhD3nHlE_YhOlC4bNMr-h1IB92zq-oXR6L6iVA0hxjNIlQ?e=nVabBp>

Baidu:

<https://pan.baidu.com/s/1X_05AGpmGEJQlzmwY8V62Q>

Dropbox:

<https://www.dropbox.com/sh/j8vohu5tw91ihd2/AAC-ksnn1exRwQagVjC7dCkYa?dl=0>

Google Drive

<https://drive.google.com/open?id=1hU1J6A8w_8zM99KAh4_1YUFvbjWelwFk>

| MDM Modules | Coating | Function |
| --- | --- | --- |
| 1. Base board   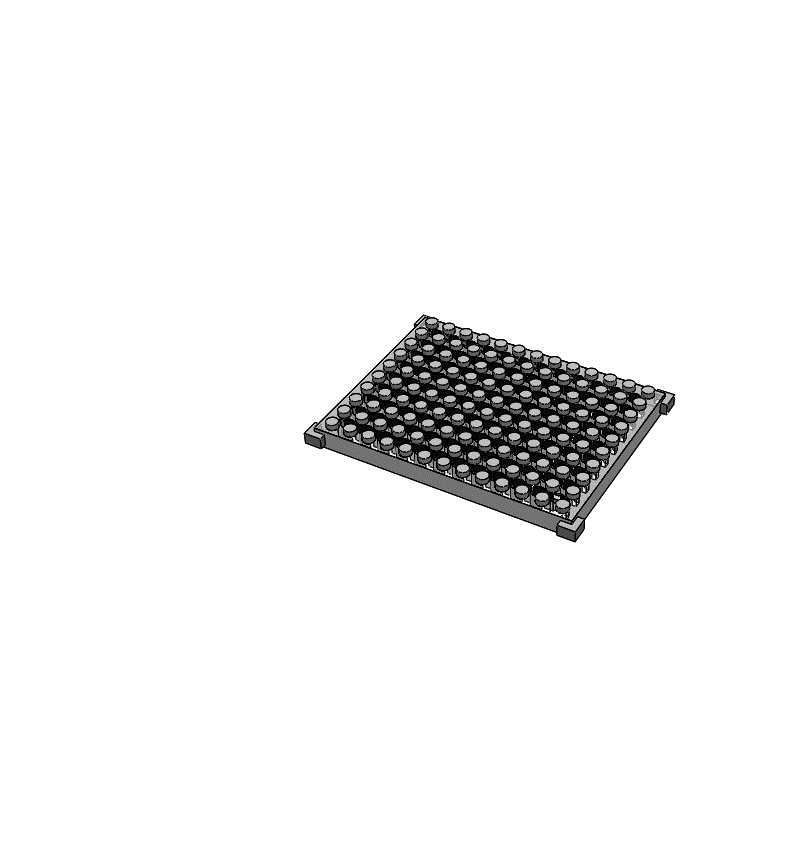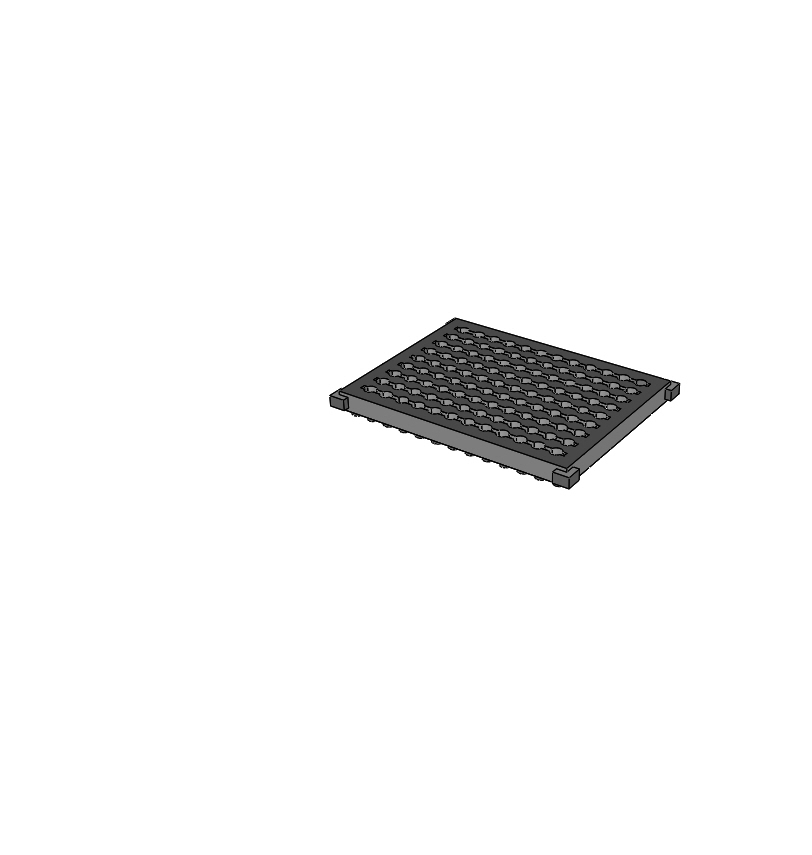 | N/A | The 67mm x 50mm base board are printed with clear resins using stereolithography (SLA). It contains 13 × 10 studs with a diameter of 3 mm. The spaces between studs were perforated to create access for the liquid dispenser. |
| 1. Glass coverslip substrate   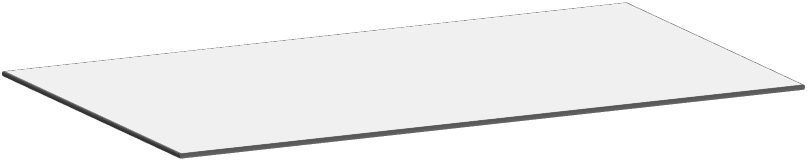 | Teflon | The 65x48 mm glass coverslips were spin-coated with 1% Teflon AF solution. It serves as the substrate where droplets sit on. |
| 1. Support frame   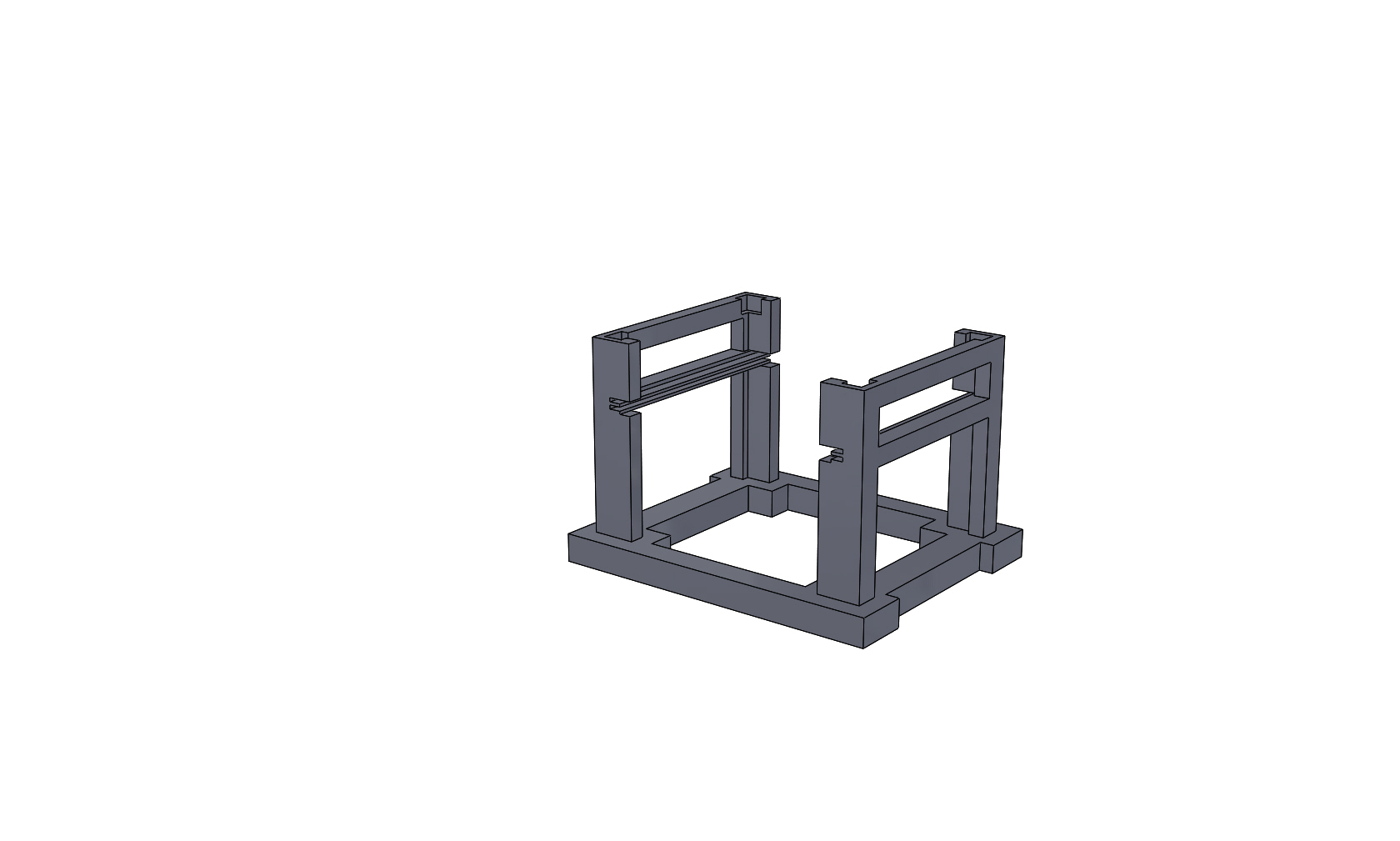 | N/A | Support holder is 3D printed with acrylonitrile butadiene styrene (ABS) using fused deposition modeling (FMD). It functions as a support for both baseboard and glass coverslip substrate. |
| 1. Particle extraction module   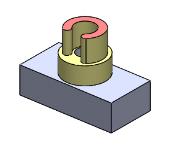 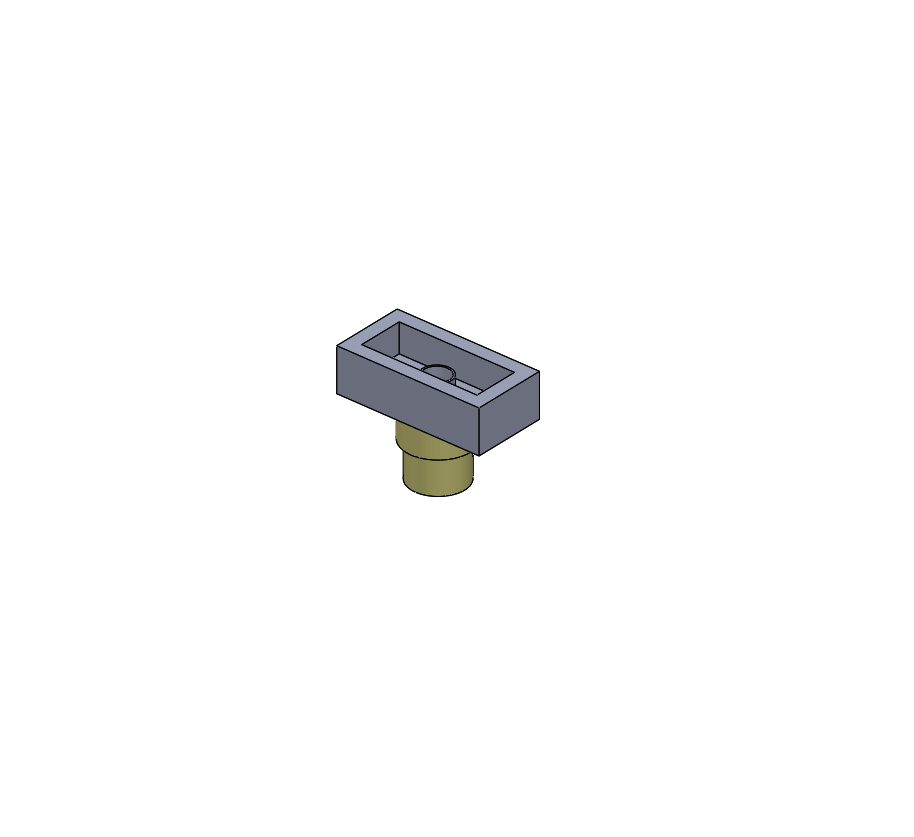 | Teflon and PDA | A Teflon coated hollow cylinder with its circular tip coated with polydopamine.  The entire droplet is anchored to the SET while the magnetic particle cluster continues moving forward until it breaks from the droplet. |
| 1. Holder module   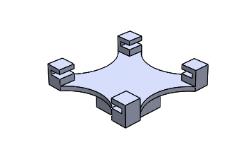 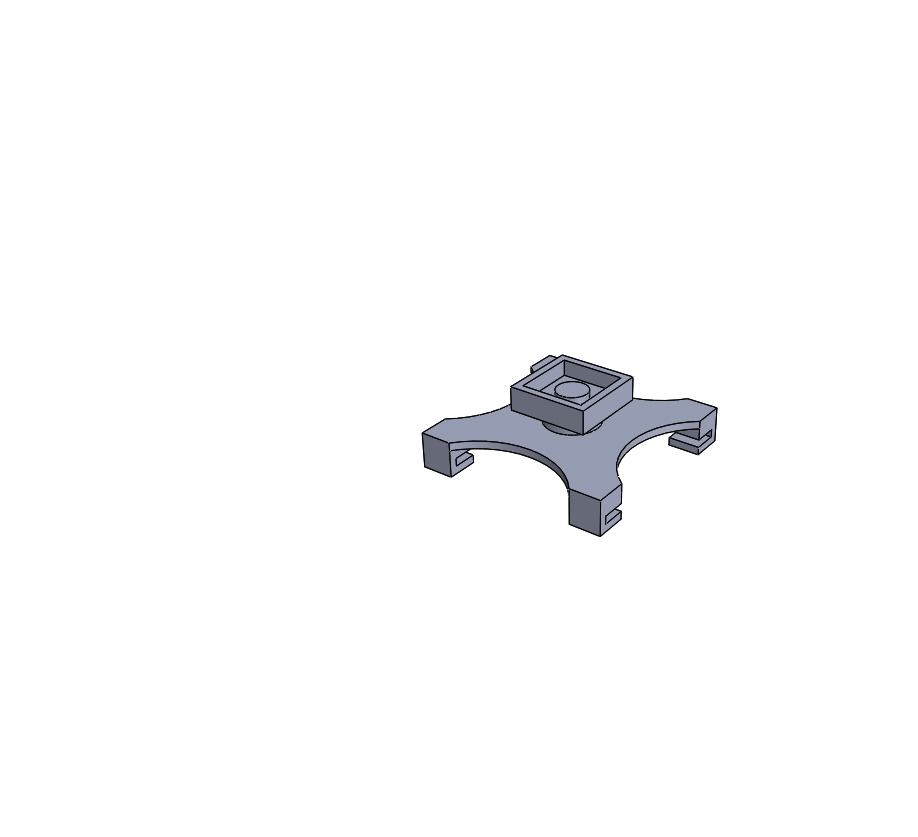 | N/A | Modified glass coverslip can slide in the holder module. The droplets are sandwiched between two pieces of glass coverslip. It can perform both particle extraction and liquid dispensing operation depending on the type of modified glass coverslip inserted. |
| 1. Liquid dispensing module   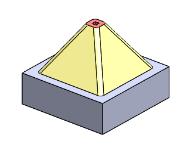 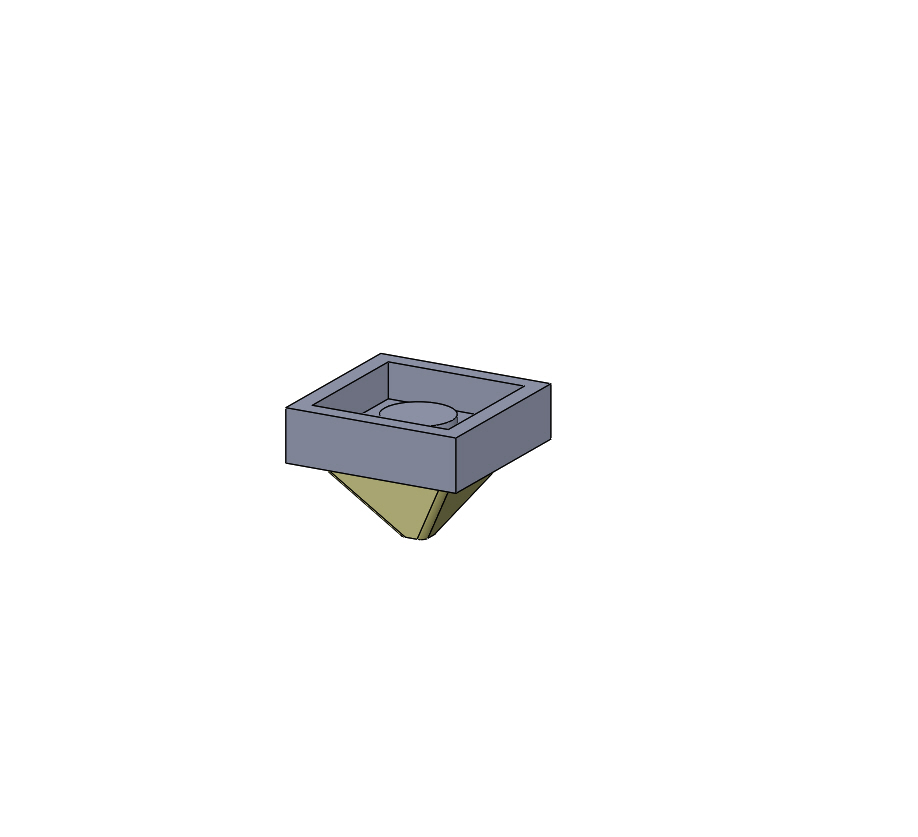 | Teflon and PDA | A pyramid with a small flat tip coated with polydopamine. Due to the small area of the SET, only a small portion of the droplet is attached to the SET while the rest pass by the module. |
| 1. Mixing module.   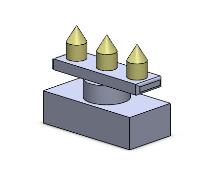 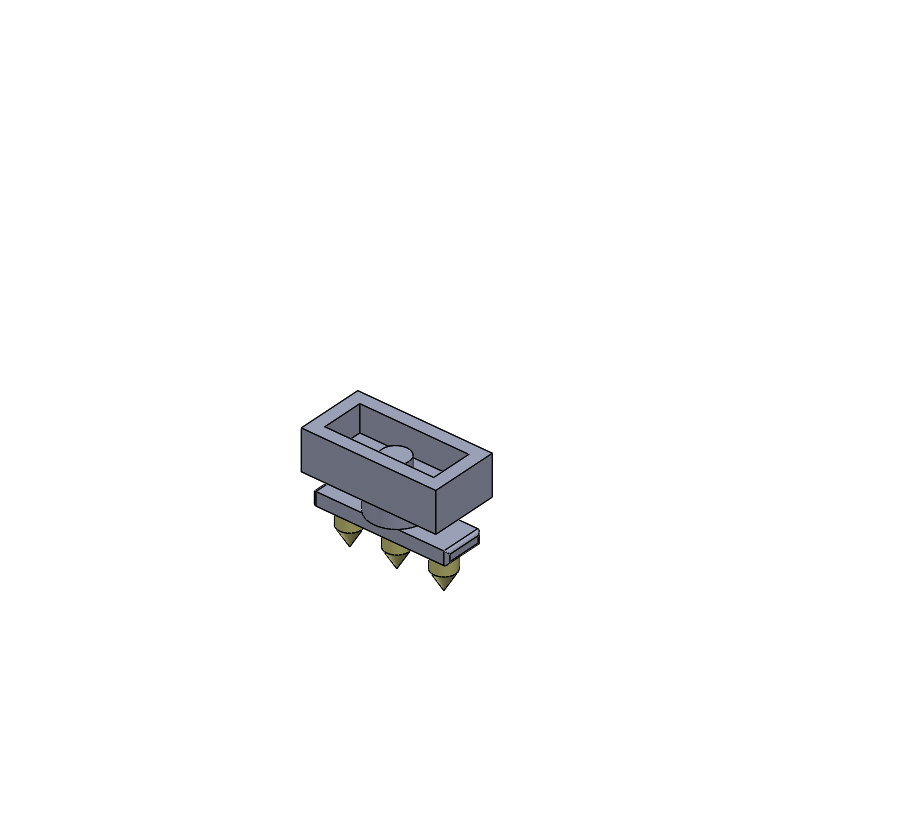 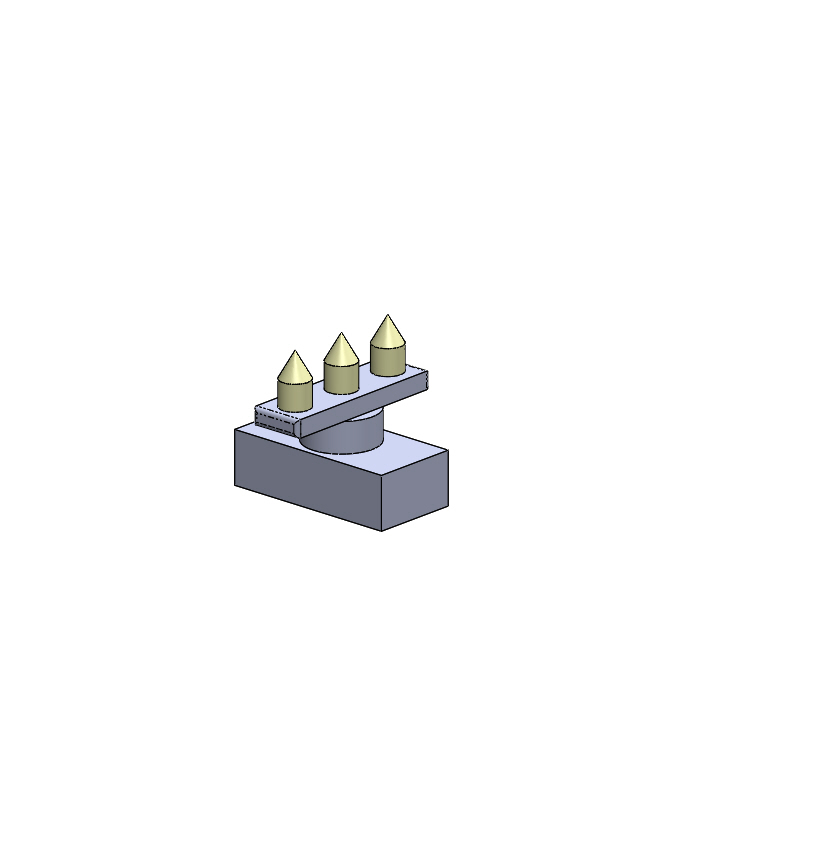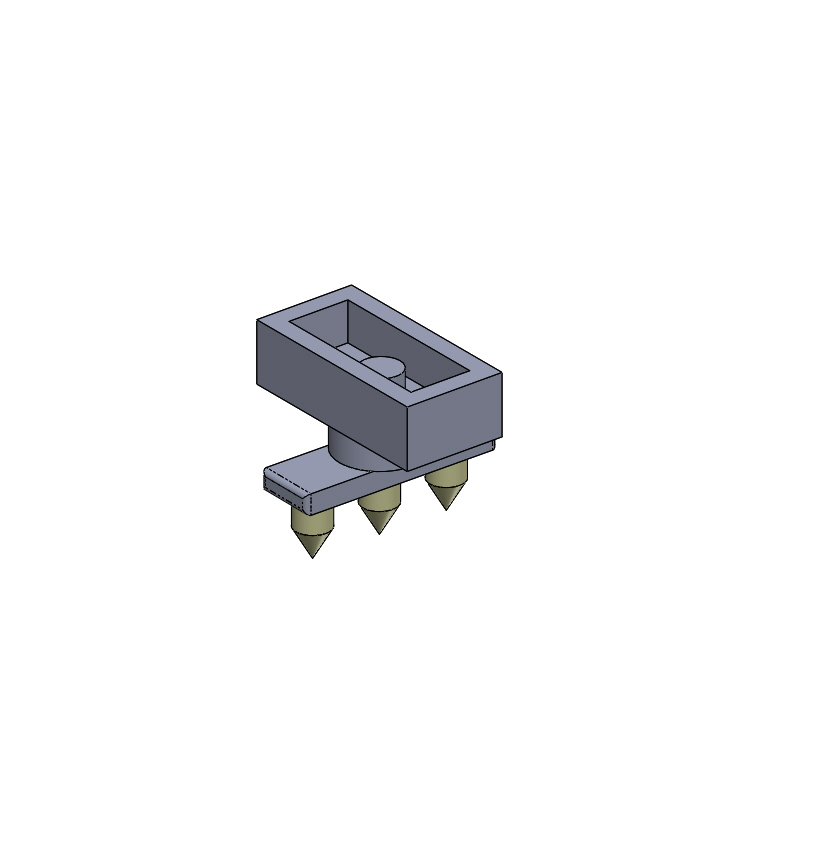 | Teflon | Three tapered pillars with a rough surface resulted from the printing process. Although the surface of the pillars is coated with Teflon, the droplet would still adhere slightly to the pillar due to the Wenzel wetting on the rough surface causing mixing in the droplet. |

**Fig. S1** Library of modular components for modular MDM.


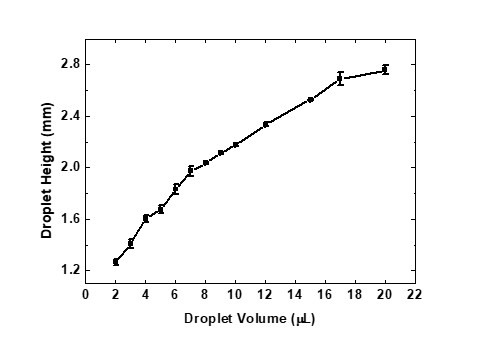


**Fig. S2** Droplet height vs droplet volume on the Teflon-coated surface.


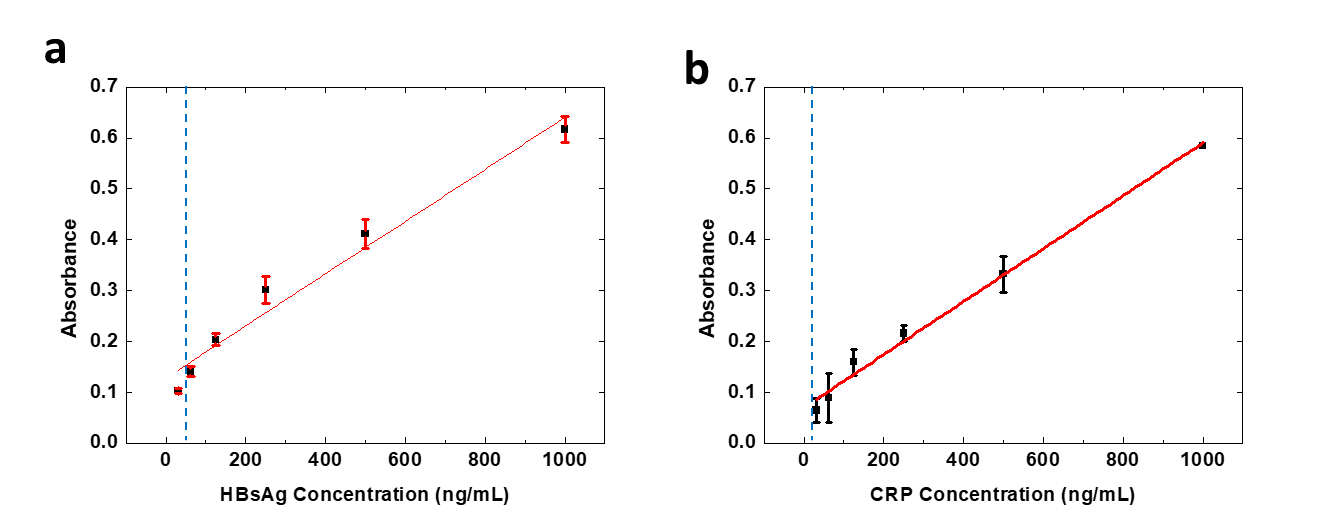


**Fig. S3** Benchmark ELISA performed in microwell plate. a) Standard curve of HBsAg. b) Standard curve of CRP. The dash lines indicate the limit of detection.

**
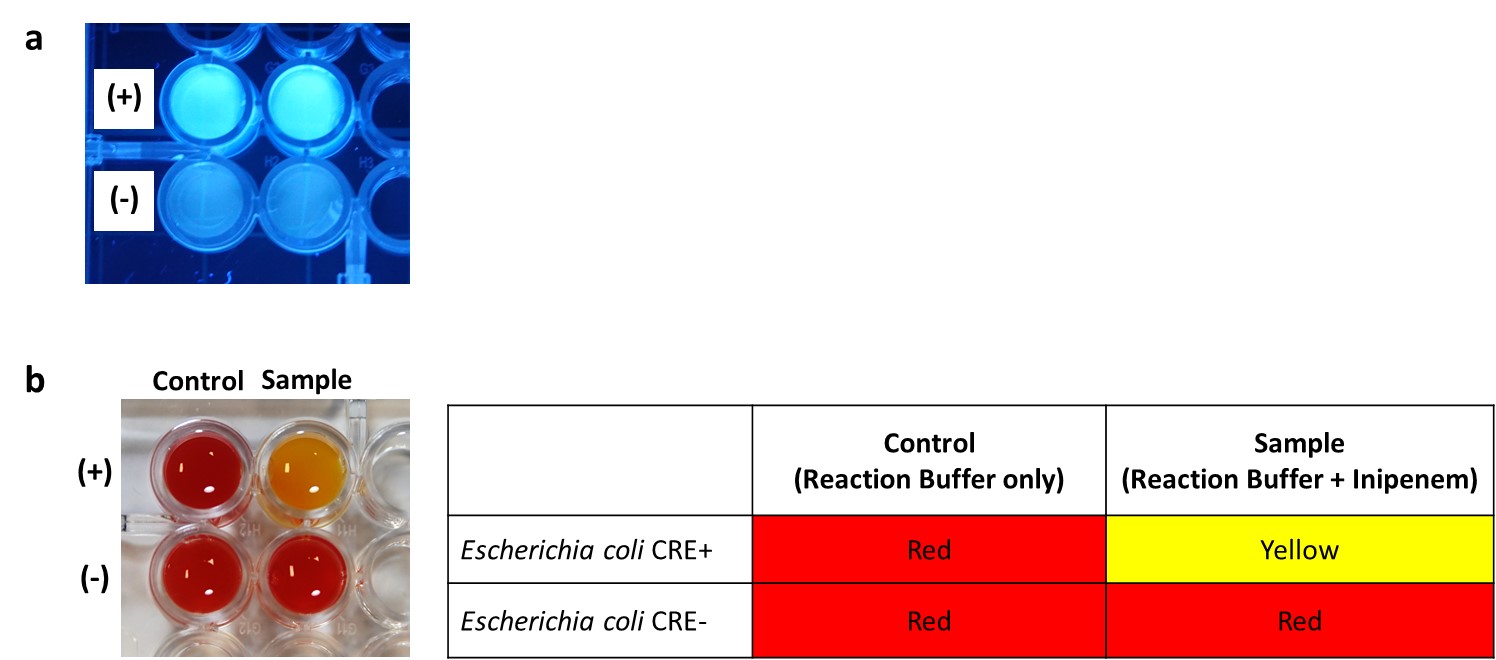
**

**Fig. S4** Benchmark phenotypical assays performed in microwell plate. a) MUG test. b) Carba NP test.


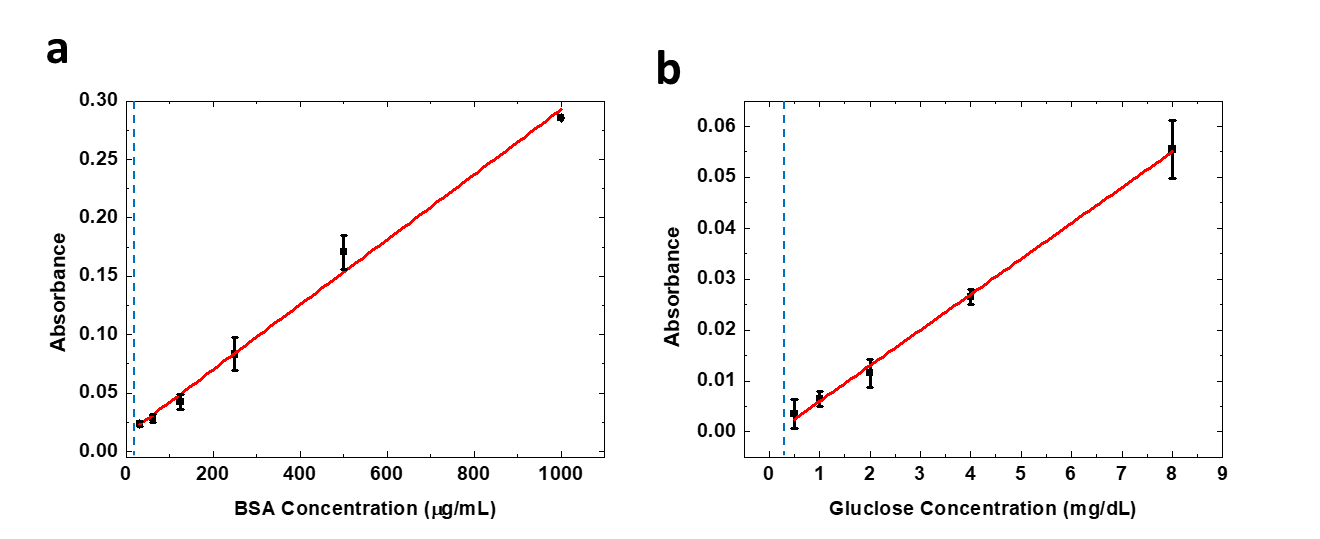


**Fig. S5** Benchmark enzymatic assays performed in microwell plate. a) Standard curve of BSA. b) Standard curve of glucose. The dash lines indicate the limit of detection.
